# Gab Docking Proteins in Cardiovascular Disease, Cancer, and Inflammation

**DOI:** 10.1155/2013/141068

**Published:** 2013-01-22

**Authors:** Yoshikazu Nakaoka, Issei Komuro

**Affiliations:** Department of Cardiovascular Medicine, Graduate School of Medicine Osaka University, 2-2 Yamadaoka, Suita, Osaka 565-0871, Japan

## Abstract

The docking proteins of the Grb2-associated binder (Gab) family have emerged as crucial signaling compartments in metazoans. In mammals, the Gab proteins, consisting of Gab1, Gab2, and Gab3, are involved in the amplification and integration of signal transduction evoked by a variety of extracellular stimuli, including growth factors, cytokines, antigens, and other molecules. Gab proteins lack the enzymatic activity themselves; however, when phosphorylated on tyrosine residues, they provide binding sites for multiple Src homology-2 (SH2) domain-containing proteins, such as SH2-containing protein tyrosine phosphatase 2 (SHP2), phosphatidylinositol 3-kinase regulatory subunit p85, phospholipase C**γ**, Crk, and GC-GAP. Through these interactions, the Gab proteins transduce signals from activated receptors into pathways with distinct biological functions, thereby contributing to signal diversification. They are known to play crucial roles in numerous physiological processes through their associations with SHP2 and p85. In addition, abnormal Gab protein signaling has been linked to human diseases including cancer, cardiovascular disease, and inflammatory disorders. In this paper, we provide an overview of the structure, effector functions, and regulation of the Gab docking proteins, with a special focus on their associations with cardiovascular disease, cancer, and inflammation.

## 1. Introduction

The mammalian Grb2-associated binder (Gab) proteins are homologs of *Drosophila* DOS (Daughter Of Sevenless) and *Caenorhabditis elegans* SOC-1 (Suppressor Of Clear). These proteins define a family of docking proteins closely related to the insulin receptor substrate (IRS-1, IRS-2, IRS-3), fibroblast growth factor substrate (FRS2), linker of T cell (LAT), and downstream of kinase (Dok) families [1]. In contrast to adaptor proteins such as growth factor receptor bound protein 2 (Grb2) and Shc, which are usually smaller and often function as a molecular bridge between two proteins in the assembly of larger protein complexes, docking proteins contain a membrane-targeting region at the N-terminus, binding sites for src homology 3 (SH3) domain-containing proteins, and multiple tyrosine phosphorylation sites that, when phosphorylated, function as binding sites for the src homology 2 (SH2) domains of a variety of effectors. Consequently, the docking proteins are significantly larger than adaptor proteins. In addition, docking proteins usually contain one or more moieties that mediate their recruitment to plasma membranes through protein-protein or protein-lipid interactions. Their multiple functional domains and large molecular size reflect the docking proteins' function as a platform for the assembly of signaling subsystems. Since there have been several excellent general reviews on Gab proteins to date [1–4], here we will focus on the role of Gab docking proteins in cardiovascular and inflammatory disorders.

## 2. Identification of Gab Family Docking Proteins

Gab1, the first of the three mammalian *gab* genes cloned to date, was originally identified as a Grb2-binding protein from a human glial tumor expression library and found to undergo tyrosine phosphorylation in response to stimulation by epidermal growth factor (EGF) and insulin [5]. It was also isolated as a c-Met-receptor interacting protein in a yeast two-hybrid screen and as the major tyrosine-phosphorylated protein in cells transformed by the *Tpr-Met* oncogene [6, 7]. Gab2 was cloned as a binding protein and a substrate of the SH2 domain-containing protein tyrosine phosphatase (SHP2) [8–10]. The *Gab3* cDNA was cloned with the aid of the genome sequencing project, using a search strategy based on sequence similarities to Gab1 [11]. Although a putative *Gab4* gene has been found in the human genome database, its expression pattern, signaling mechanism, and functional roles have not been characterized to date.

DOS is the only Gab homolog in *Drosophila*. It was identified as a potential substrate for the product of *Corkscrew* (*Csw*) [12], the *Drosophila* SHP2 ortholog, and independently in a screen for mutants that suppress the rough-eye phenotype of a hyper-activated *sevenless* allele [13]. SOC-1, the *C. elegans* homolog, was found in a screen for suppressors of hyperactive Egl-15 (an FGF receptor ortholog) signaling [14]. 

## 3. Molecular Structure, Recruitment, and Phosphorylation of Gab Docking Proteins 

### 3.1. Molecular Structure

**Figure 1:**
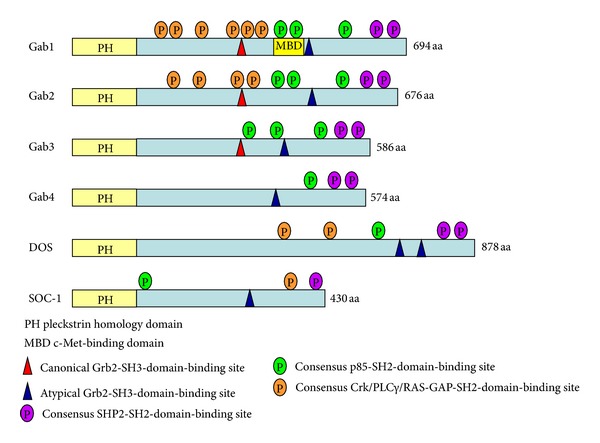
Schematic structures of Gab family docking proteins. Shown are the schematic domain structures of three human Gab proteins (Gab1–3), the putative human Gab4 protein, *Drosophila* DOS, and *C. elegans* SOC-1. All Gab proteins consist of a highly conserved N-terminal PH domain that is involved in membrane targeting. The central proline-rich regions mediate the association with SH3 domain-containing adaptor proteins such as Grb2. Consensus binding motifs favored by various SH2 domain-containing proteins such as SHP2, p85, Crk, and PLC*γ* are indicated.

All Gab docking proteins share a highly conserved N-terminal Pleckstrin homology (PH) domain, proline-rich segments in the central region, and multiple tyrosine residues within the potential binding motifs favored by various SH2 domain-containing signaling proteins (Figure 1) [1–4]. Mutagenesis and *in vitro* binding assays have demonstrated that a number of signaling molecules interact with Gab docking proteins (Figure 1). 

### 3.2. Recruitment

Gab docking proteins utilize several different mechanisms to regulate their subcellular localization. First, the PH domain enables Gab proteins to translocate to plasma membrane patches enriched in phosphatidylinositol 3,4,5-triphosphate (PIP3), a product of phosphatidylinositol-3 kinase (PI3K) [15–18]. Besides the PH domain, Gab docking proteins use at least two additional mechanisms for their recruitment to activated plasma membrane-associated receptors. The first mechanism has been demonstrated only for the interaction between Gab1 and c-Met (the receptor for hepatocyte growth factor; HGF) [7]. A region in Gab1 (amino acids 450–532), termed the c-Met binding domain (MBD), interacts directly with the tyrosyl-phosphorylated c-Met in response to stimulation with HGF [7, 19–21]. Both the activated kinase domain of c-Met and the MBD in Gab1 are involved in this direct interaction [19, 20]. The minimal amino acid sequence sufficient for the direct interaction between Gab1 and c-Met, termed the c-Met binding sequence (MBS), consists of 16 amino acids (486–501) [19, 20]. Since no other Gab docking proteins contain the MBS [22, 23], Gab2 interacts with activated receptors via the adaptor protein Grb2, which is also utilized as a secondary mechanism by the c-Met receptor to associate indirectly with Gab1. The importance of this indirect recruitment was revealed in knockout mice expressing a Gab1 mutant incapable of binding with Grb2: the phenotype was lethal [21].

### 3.3. Phosphorylation

Gab-mediated signal transduction is regulated by the site-specific tyrosine phosphorylation of the Gab proteins. Phosphorylated tyrosine residues provide docking sites for the SH2-domains of SHP2, the Crk adaptor protein, phospholipase C (PLC) *γ*, and the regulatory subunit of PI3K, p85 [2–4]. By recruiting various effectors with SH2 domains, Gab proteins not only promote signal transduction but also translate the receptor-evoked signals into distinct biological properties. Therefore, Gab family proteins function as a signaling platform for an entire signaling subsystem.

The best-characterized effector signaling pathway of Gab proteins is transmitted via the protein tyrosine phosphatase SHP2. SHP2 has two tandem N-terminal SH2 domains, which confer autoinhibition of the C-terminal phosphatase domain [24]. All mammalian Gab proteins, as well as the *Drosophila* DOS and *C. elegans* SOC-1, bind SHP2 (or its homologs), suggesting that the recruitment of SHP2 is an evolutionarily conserved feature of Gab family proteins [24]. Most Gab proteins contain two SHP2 binding sites, which act as a biphosphoryl tyrosine activation motif (BTAM) and bind both SH2 domains, which releases SHP2's autoinhibition [24, 25]. Therefore, SHP2 binding partners, including Gab proteins, may act not only as signaling platforms, but also as allosteric activators. 

The functional significance of the Gab-SHP2 interaction has been extensively studied using mutants of Gab family proteins unable to bind SHP2 or its homologs. Mutant DOS bearing a Y to F mutation at either of the two CSW-binding sites is nonfunctional, and Sevenless signaling cannot rescue the lethal phenotype associated with *DOS* loss-of-function mutations [26, 27]. A Gab1 mutant that is unable to bind SHP2 fails to transduce the signal for c-Met-dependent morphogenesis in MDCK cells and blocks the activation of extracellular signal-regulated kinase 1/2 (ERK1/2), MAP kinase upon stimulation with epidermal growth factor (EGF), HGF, or lysophosphatidic acid (LPA) [23, 25, 28, 29]. In endothelial cells, the recruitment of SHP2 to Gab1 not only regulates vascular endothelial growth factor- (VEGF-) induced migration, but also contributes to HGF-induced migration [30–32]. We also found that the Gab1-SHP2 interaction is involved in the activation of extracellular signal-regulated kinase 5 (ERK5) in gp130-dependent cardiomyocyte hypertrophy [33, 34]. In addition, in certain cellular circumstances, the Gab-SHP2 complex positively regulates other downstream pathways, such as c-Kit-induced Rac activation and *β*1-integrin-induced PI3K activation [35, 36]. 

These findings demonstrate that SHP2 is a crucial positive modulator for the activation of ERK1/2. Although the molecular mechanism underlying why the recruitment of SHP2 by Gab1 is required for the full activation of ERK1/2 is still not completely understood, two possible mechanisms have been proposed. First, SHP2 may dephosphorylate the recruitment site for the Src-inactivating kinase Csk on the transmembrane glycoprotein PAG/Cbp and paxillin, resulting in the enhanced activation of Src family kinases [37, 38]. Second, SHP2 may dephosphorylate the binding site for p120Ras-GAP on the activated receptors for EGF and on Gab1, thus inactivating the Ras-dependent signaling pathway [37, 39].

## 4. Physiological Functions of Gab Proteins Revealed by Global Knockout or Knock-In Mice

The presence of multiple *gab* genes in mammals suggests that the function of each Gab protein may be specialized or restricted to certain pathways or tissues. On the other hand, these gene products may be functionally redundant. Extensive analyses of the expression level of the *gab* genes by northern blot and RT-PCR have shed some light on this issue [5, 7, 8, 11]. Gab1 shows the broadest expression and greatest abundance: it is found in almost all tissues examined, including the brain, heart, liver, lung, kidney, pancreas, spleen, thymus, and uterus of the adult mouse, and is expressed at early developmental stages, such as in ES cells [11]. Although Gab2's expression is relatively weak in most tissue samples, compared with Gab1, it is abundantly expressed in hematopoietic progenitor cell lines, such as BAF3 and FDC-P1 [5, 7, 8, 11]. The expression of Gab3 is also confined to the hematopoietic system [5, 7, 8, 11]. Thus, the three mammalian *gab* genes have unique but overlapping expression patterns. 

Consistent with Gab1's early and broad expression during development, Gab1-knockout (Gab1^−/−^) mice die *in utero* between embryonic days (E) 13.5 and 18.5 with developmental defects in the heart, placenta, skin, and skeletal muscle [40, 41]. In line with the close relationship between Gab1 and c-Met, Gab1^−/−^ mice phenocopy most of the phenotypes of HGF- and c-Met-knockout mice, such as early embryonic lethality with placental defects, reduced liver size, and defects in the migration of muscle precursor cells [40, 41]. 

Gab1 knock-in mice carrying mutations in the SHP2 binding site show defects in muscle and placental development presumably directed by HGF/c-Met signaling, demonstrating a specific role for the Gab1-SHP2 complex in the migration of muscle progenitor cells [21]. Consistent with these findings, we found that the myogenic differentiation of C2C12 cells induced by IGF-1 or low-serum conditions was strongly enhanced by the adenovirus-mediated overexpression of a mutated Gab1 (Gab1^ΔSHP2^) incapable of binding SHP2, but inhibited by the overexpression of wild-type Gab1 [42]. This result suggests that Gab1 negatively regulates myogenic differentiation through its association with SHP2. Taken together, these findings suggest that Gab1 plays a key role not only in the inhibition of myogenesis, but also in the maintenance of the undifferentiated state of mesenchymal cells, effected through the activation of SHP2. On the other hand, Gab1 knock-in mice carrying mutations in the p85 binding site show defects in EGF receptor-mediated embryonic eyelid closure and keratinocyte migration [21], and knock-in mice expressing a Gab1 mutant lacking the Grb2 binding sites display an embryonic lethal phenotype and defects in liver, placenta, and craniofacial development [21]. These results support the idea that Gab1 induces different biological responses through the recruitment of distinct effectors* in vivo*. 

In contrast, Gab2-knockout (Gab2^−/−^) mice are viable, generally healthy, and have an apparently normal life span. Although Gab2 was initially believed to be essential for the development of various hematopoietic lineages through its association with SHP2 [43], steady state hematopoiesis is largely normal in Gab2^−/−^ mice [44, 45]. However, Gab2^−/−^ mice exhibit a drastic phenotype in mast cell functioning [44]. Mast cells are major players in allergic responses, and Gab2^−/−^ mice have severe defects in their response to passive allergic challenge; their mast cells display defects in degranulation and cytokine gene expression in response to the activation of Fc*ε*RI, the high-affinity IgE receptor. The defective activation of Gab2^−/−^ mast cells is ascribed mainly to their failure to induce PI3K activation. Furthermore, Gab2^−/−^ mice show decreased numbers of mast cells in various tissues, including the skin and stomach, because of weakened c-Kit signaling [45]. These findings suggest that Gab2, which is often upregulated in inflammatory disease, might be an important target for novel therapies against inflammation and allergy [46]. 

Gab2^−/−^ mice also exhibit an osteopetrotic phenotype that is attributed to the role of Gab2 in regulating RANK- (receptor activator of nuclear factor-*κ*-B-) dependent signaling [47]. Gab2 associates with RANK and mediates the RANK-induced activation of NF-*κ*B, AKT, and JNK. Bone homeostasis is determined by an intricate balance between the anabolic action of mesenchymal osteoblasts and the catabolic action of osteoclasts. Consistent with Gab2's pivotal role in the differentiation of a variety of hematopoietic lineages [43, 45], Gab2^−/−^ mice exhibit defective osteoclast differentiation, resulting in decreased bone resorption and a subsequent systemic increase in bone mass [47]. In addition, Gab2 has a crucial role in the differentiation of human progenitor cells into osteoclasts [47].

To dissect the Gab2-dependent signaling pathways required for the degranulation of mast cells *in vivo*, Nishida et al. established knock-in mice that express Gab2 mutated at the binding sites for either the PI3K regulatory subunit p85 or SHP2 [48]. They found that both binding sites of Gab2 are required for degranulation and the anaphylaxis response, but not for cytokine production or contact hypersensitivity. Interestingly, the PI3K, but not the SHP2, binding site turned out to be important for granule translocation during degranulation. In particular, the Fyn/Gab2/PI3K-signaling pathway activates a small GTPase, ADP-ribosylation factor (ARF)1, which regulates granule translocation. These results indicated that Fyn/Gab2/PI3K/ARF1-signaling is specifically required for granule translocation and the anaphylaxis response in mast cells [48].

No specific role has been identified to date for Gab3. Gab3^−/−^ mice are healthy and viable, and no obvious phenotype was detected in Gab3^−/−^ macrophages, despite the strong upregulation of this protein during macrophage differentiation [49].

## 5. Physiological Functions of Gab Proteins Revealed by Conditional Knockout Mice

### 5.1. The Roles of Gab Proteins in Cardiomyocytes

Because the Gab1^−/−^ phenotype is embryonically lethal in mice, several groups, including ours, have created conditional Gab1-knockout mice, to determine its physiological functions in adulthood [50–53]. Gab1 is exclusively expressed in the heart from E10.5 to 13.5 [40], indicating that it might have a specific role in the heart. Therefore, we created cardiomyocyte-specific Gab1-knockout (Gab1CKO) mice, but these mice are viable and display no obvious cardiac phenotypes [52]. 

**Figure 2:**
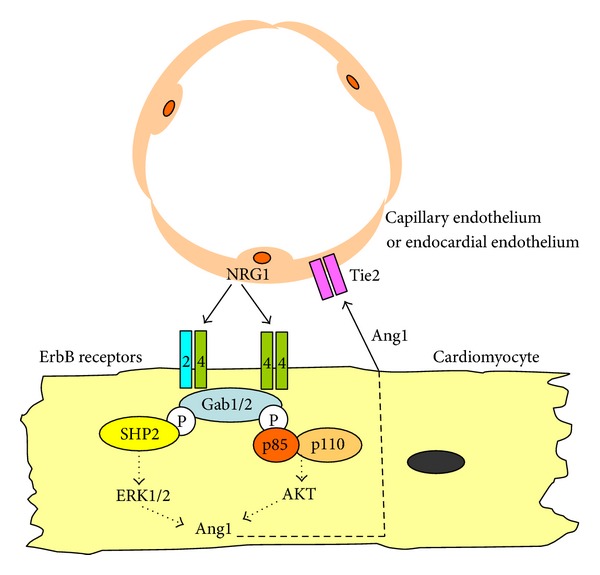
Schematic illustration of the roles of Gab docking proteins in the myocardium. Neuregulin-1 (NRG-1) shed from the capillary or endocardial endothelium in the heart activates the ErbB receptors expressed on cardiomyocytes, leading to the tyrosine phosphorylation of Gab1 and Gab2 and subsequent activation of ERK1/2 and AKT. NRG-1/ErbB-Gab1/Gab2 signaling in the myocardium is directly required for the postnatal maintenance of myocardial function. Furthermore, NRG-1/ErbB-Gab1/Gab2 signaling indirectly contributes to the postnatal stabilization of capillary or endocardial endothelium via the upregulation of angiopoietin-1 (Ang1). Ang1 derived from myocardium activates the Tie2 receptor, which is expressed on the cardiac endothelial cells.

Since Gab1 and Gab2 are expressed in cardiomyocytes, we hypothesized that Gab2 might complement the loss of Gab1. We therefore created cardiomyocyte-specific Gab1/Gab2 double-knockout (DKO) mice by crossing Gab1CKO mice with Gab2^−/−^ mice [52]. Although the DKO mice were viable, they showed a high postnatal mortality rate with marked ventricular dilatation and reduced contractility. In addition, the DKO mice showed remarkable pathological phenotypes including endocardial fibroelastosis and a large number of abnormally dilated coronary vessels in the ventricles. Neuregulin-1 (NRG-1) and ErbB receptors, including ErbB2 and ErbB4, comprise an important signaling pathway for heart development and the maintenance of heart function in adulthood. The NRG-1-induced activation of ERK1/2 and AKT were observed in the hearts of control, Gab1CKO, and Gab2^−/−^ mice, but not of DKO mice. These results suggest that Gab1 and Gab2 share a critically redundant role in NRG-1-dependent signaling in cardiomyocytes (Figure 2).

To determine the effects of the DKO on gene expression, we performed a DNA microarray analysis of cardiac tissues, and found that NRG-1 upregulates the expression of the endothelium-stabilizing factor, angiopoietin-1 (Ang1), in the control mice, but not in the DKO mice [52]. Conventional Ang1-knockout mice show impaired development of myocardial trabeculae and vessel maturation [54], which are quite similar to the pathological abnormalities in the hearts of the DKO mice. Furthermore, the expression patterns of NRG-1 and ErbB are almost mirrored by those of Ang1 and Tie2, in the heart, suggesting that these two signaling pathways influence each other like a paracrine signaling circuit in the cardiac microenvironment [55]. These results suggest that the contributions of Gab1 and Gab2 to the crosstalk between NRG-1/ErbB and Ang1/Tie2 signaling are required for the maintenance of heart function (Figure 2). 

### 5.2. The Role of Gab1 in Angiogenesis, Vascular Inflammation, and Atherosclerosis

**Figure 3:**
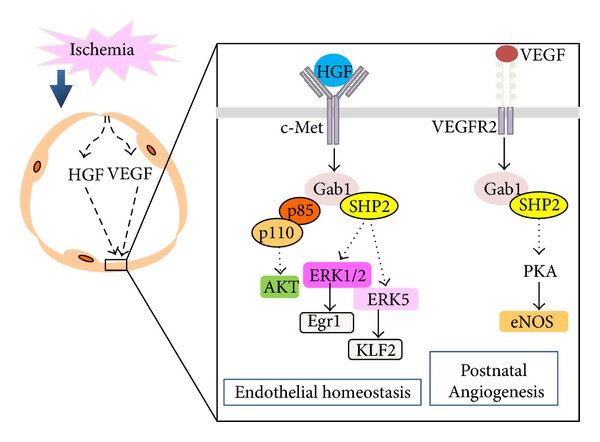
Schematic illustration of the role of Gab1 in postnatal angiogenesis and endothelial homeostasis. Hypoxic tissues secrete growth factors, such as HGF and VEGF, which stimulate their specific receptors on endothelial cells (inset). The activation of c-Met receptors leads to the tyrosine phosphorylation of Gab1 and thereby to the subsequent complex formation of Gab1 with both SHP2 and p85. Whereas the formation of the Gab1-SHP2 complex is required for the activation of ERK1/2 and ERK5, the Gab1-p85 complex is essential for the activation of AKT in response to HGF. The ERK1/2 and ERK5 pathways contribute to the upregulation of early growth response 1 (Egr1) and Kruppel-like factor 2 (KLF2). On the other hand, activation of the VEGFR2 receptors leads to the tyrosine phosphorylation of Gab1, and to the subsequent formation of the Gab1-SHP2 complex, which causes the activation of protein kinase A (PKA) and endothelial nitric oxide synthase (eNOS). Collectively, current findings indicate that Gab1 is an essential component of postnatal angiogenesis after ischemia.

Angiogenesis, the process of new blood vessel formation, is involved in many pathological settings, including ischemia, atherosclerosis, diabetes, and cancer [56]. It has been reported that Gab1 has a role in vascular endothelial growth factor- (VEGF-) dependent signaling in *in vitro* experiments using endothelial cells (ECs) [30, 31]. To reveal the *in vivo* role of Gab proteins in angiogenesis, we created endothelium-specific Gab1 knockout (Gab1ECKO) mice [32]. The Gab1ECKO mice are viable and do not show any obvious defects in vascular development. We then subjected Gab1ECKO and Gab2^−/−^ mice to hindlimb ischemia (HLI) induced by unilateral femoral artery ligation. Intriguingly, impaired blood flow recovery and necrosis in the operated limb was observed in all the Gab1ECKO mice, but not in the control (wild-type) or Gab2KO mice. In human ECs, we compared the effects of several angiogenic growth factors and found that HGF induces the most prominent tyrosine phosphorylation of Gab1 and the greatest subsequent complex formation of Gab1 with both SHP2 and p85 [32]. The Gab1-SHP2 complex was required for both the HGF-induced migration and proliferation of ECs via the ERK1/2 pathway and the HGF-induced stabilization of ECs via ERK5. The Gab1-p85 complex also regulated the migration of ECs after HGF stimulation, and it regulates the activation of AKT [32]. A microarray analysis of HGFs effects on gene expression in ECs demonstrated that it upregulates angiogenesis-related genes such as *Kruppel-like factor 2 *(*KLF2*) and *early growth response *1 via the Gab1-SHP2 complex in human ECs (Figure 3) [32]. Furthermore, gene transfer of VEGF, but not HGF, improved the blood flow recovery and ameliorated the limb necrosis after HLI in the Gab1ECKO mice [32]. These results suggest that Gab1 is essential for postnatal angiogenesis after ischemia via PI3K HGF/c-Met signaling (Figure 3). 

At the same time as our study, two other groups reported results on postnatal angiogenesis in Gab1ECKO mice using the HLI model [57, 58]. Whereas Zhao et al. reported that endothelial Gab1 is essential for HGF-dependent postnatal angiogenesis, a finding almost identical to ours [58], Lu et al. reported that Gab1 regulates postnatal VEGF-dependent angiogenesis through the protein kinase A- (PKA-) endothelial NOS (eNOS) pathway [57]. Together, these findings provided by three independent groups show that Gab1 is a crucial signal transducer that unites the HGF-dependent and VEGF-dependent signaling and angiogenesis in endothelial cells (Figure 3) [32, 57, 58].

Since the above findings led us to hypothesize that Gab1 might have a role in endothelial homeostasis, we intercrossed the Gab1ECKO mice with apolipoprotein E (ApoE) knockout (ApoEKO) mice. Six-month-old male ApoEKO/Gab1ECKO and littermate control (ApoEKO) mice were treated with angiotensin II (AngII) via an osmotic infusion minipump for 4 weeks. After the AngII treatment, the ApoEKO/Gab1ECKO mice showed significantly exacerbated atherosclerosis and aneurysm formation compared with control mice [59]. The production of proinflammatory cytokines in the aorta was also significantly greater in the ApoEKO/Gab1ECKO than in the control mice. Furthermore, the expression levels of KLF2 and KLF4, key transcription factors for endothelial homeostasis, were significantly reduced in the aortic endothelium of the ApoEKO/Gab1ECKO mice compared with the control mice [59, 60]. Consistent with the reduced expression of KLF2 and KLF4, both vascular cell adhesion molecule-1 (VCAM-1) expression and macrophage infiltration of the aortic walls were enhanced in ApoEKO/Gab1ECKO mice compared with the control mice [59, 60]. Taken together, these findings show that endothelial Gab1 protects the endothelium from AngII-dependent vascular inflammation and atherosclerosis in the *ApoE*-null background, presumably in association with the downregulation of KLF2 and KLF4 [59].

### 5.3. The Role of Gab1 in Liver Regeneration

Liver regeneration is a rapid and concerted response to injury, in which growth factor-evoked intracellular signals lead to the activation of various transcriptional factors, DNA synthesis, and hepatocyte proliferation. Liver-specific Gab1 knockout (LGKO) mice exhibit defective liver regeneration after a two-thirds partial hepatectomy [50]. The defects in LGKO mice may be ascribed to the decreased proliferation of hepatocytes, due to the decreased activation of ERK1/2 and attenuated upregulation of immediate-early genes, such as c-fos, c-jun, and c-myc, after liver injury [50]. Interestingly, liver-specific SHP2-knockout mice phenocopy the defective liver regeneration of LGKO mice after partial hepatectomy, suggesting that Gab1 plays a critical role in liver regeneration via its association with SHP2 [50]. In addition, Gab1 negatively regulates the hepatic insulin-induced activation of AKT via the ERK1/2-mediated phosphorylation of IRS-1 on Ser612 [51]. Therefore, Gab1 is required not only for liver regeneration but also for the negative regulation of insulin-mediated hepatic glucose homeostasis. 

### 5.4. The Roles of Gab Proteins in Bone Homeostasis

The analysis of Gab2^−/−^ mice shows that Gab2 couples RANK to the downstream signaling essential for osteoclastogenesis, and that Gab2 has a negative regulatory role in osteoblast differentiation [47, 61]. In contrast, osteoblast-specific Gab1-knockout mice display a low-bone-turnover osteopenic phenotype at 2 months of age, demonstrating an essential role for Gab1 in osteoblast functioning [53]. These results indicate that Gab1 and Gab2 have distinct functions in the maintenance of bone homeostasis: Gab1 in osteoblasts and Gab2 in osteoclasts.

## 6. Gab Proteins in Human Cancers

Gab proteins have been implicated in several hematological neoplasias and solid cancers, although only a few mutations have been reported in human Gab proteins to date. It is currently established that Gab proteins promote tumorigenesis by functioning as “accomplices” of certain oncoproteins or by amplifying signaling upon the Gab proteins' overexpression. 

The chromosomal 11q13-14 locus containing the Gab2 gene is amplified in breast, ovarian, and gastric cancers and in acute myeloid leukemia (AML) [62–65]. Gab2 is overexpressed in estrogen receptor-positive cells [66], and a subset of breast cancers is driven by Gab2 overexpression coupled with RTK ErbB2 (also known as Neu or HER2) receptor signaling [62]. Consistent with these clinical results, Neel's group demonstrated that in the cultured human mammary epithelial cell line MCF-10A, the coexpression of wild-type Gab2, but not Gab2^ΔSHP2^ (incapable of binding SHP2) with ErbB2/Neu/HER2 resulted in an invasive growth phenotype [62]. They also revealed that NeuNT-transgene-evoked mammary tumorigenesis is potentiated in MMTV-Gab2 transgenic mice and attenuated in Gab2-deficient mice [62]. Similarly, Gab2's overexpression can potentiate metastatic melanomas [67]. Furthermore, myeloid progenitors from Gab2^−/−^ mice are resistant to transformation by Bcr-Abl, indicating that Gab2 is required to sustain the leukemogenesis evoked by this oncogenic fusion protein in a model of chronic myelogenous leukemia (CML) [68]. The phosphorylation of Y177 within the Bcr moiety results in the recruitment of the Grb2-Gab2 complex and the activation of downstream signaling via SHP2 and PI3K, which is essential for the cancer cells' enhanced proliferation and survival [68]. These results suggest that the Grb2-mediated recruitment of Gab2 to the oncogenic fusion protein Bcr-Abl is a critical event for the induction of a CML-like disease. Gab2 is also important in the progression of other hematological neoplasias, such as juvenile myelomonocytic leukemia (JMML), acute myelocytic leukemia (AML), and acute lymphoblastic leukemia (ALL) [65, 69]. 

That Gab1 plays a role in tumorigenesis is implied by its strong relationship with c-Met receptor signaling, since c-Met is activated, mutated, or overexpressed in a wide range of cancers [19, 70, 71]. Gab1 is also implicated as a mediator of EGFR-signaling-induced tumorigenesis in glioblastomas and intestinal adenomas [72, 73].

The elucidation of this direct linkage between Gab proteins and human cancers may contribute to the development of novel anticancer drugs in the future.

## 7. Gab Proteins in Human Cardiovascular Diseases

The neuro-cardiofacial-cutaneous (NCFC) syndromes consist of neurofibromatosis (NF), Noonan syndrome (NS), LEOPARD (multiple lentigines, electrocardiographic conduction abnormalities, ocular hypertelorism, pulmonary stenosis, abnormal genitalia, growth retardation, and sensori-neuronal deafness) syndrome (LS), Costello syndrome, and cardiofacial-cutaneous syndrome. All of these syndromes are associated with autosomal-dominant germline mutations within either the core components (Ras, B-Raf, Raf-1, MEK) of the Ras-ERK1/2 pathway or its modulators (NF1, SHP2, SOS, and Spred). The resulting mutant proteins exhibit abnormal activities and disturbed overall fine-tuning of the Ras-ERK1/2 pathway (and to some extent of the Ras-PI3K pathway) [74, 75]. Since the ERK1/2 pathway has a central role in both proliferation and differentiation, many processes in human development and organ maintenance are disturbed by its dysfunction, resulting in various clinical symptoms, such as a distinctive cranio-facial appearance, cardiac defects, musculocutaneous abnormalities, and mental retardation [74, 75]. Germline missense mutations in the SHP2-encoding *PTPN11* gene are seen in approximately 50% of NS cases; this observation contributed to the identification of *PTPN11* as the most common target of somatic mutations in JMML [76, 77]. The most frequent JMML-associated mutation, E76 K, confers an enhanced catalytic activity on SHP2 and requires Gab2 for the transformation of primary murine myeloid progenitors [69]. This result demonstrates that Gab2 is an essential player in JMML and suggests that NS-associated SHP2 mutants may require Gab proteins similarly, as a recruitment tool. 

Dominant-negative mutations of SHP2 are reported in LS patients, although NS patients usually carry constitutively active SHP2 mutations [78]. Intriguingly, the expression of LS-associated SHP2 mutants with reduced catalytic activity in cultured cells significantly enhances the EGF-induced association of Gab1 with p85 [79]. This result suggests that LS-associated mutations in SHP2 might potentiate abnormal PI3K activation by blocking SHP2 from dephosphorylating the p85 recruitment sites on the Gab proteins. Collectively, these studies suggest that Gab proteins might exert an important role as “accomplices” of NCFC-associated SHP2 mutants in the pathogenesis of NCFC syndromes.

## 8. Molecular Mimicry of Gab Proteins by a Bacterial Virulence Factor, CagA 

The CagA protein of the gastric pathogen *Helicobacter pylori,* a rod-shaped bacterium that infects the epithelial cells lining the stomach, has been described as functioning as a Gab-like protein [80]. The CagA protein is injected into the cytoplasm of gastric epithelial cells by the bacterium, whereupon it undergoes tyrosine phosphorylation by Src family kinases and c-Abl on E-P-I-Y-A sequence motifs present in its C-terminal region [81, 82]. Subsequently, CagA recruits SH2 domain-containing effector proteins such as SHP2 and Grb2, enabling CagA to effectively take over the signaling pathways that are normally regulated by Gab proteins. This process results in the rearrangement of actin cytoskeleton, cell scattering, and cell elongation, termed the “hummingbird” phenotype, which is reminiscent of the cellular response to Gab activation in cardiomyocytes and other cells [33, 83]. 

CagA has been categorized as a Gab mimic based on its ability to interact with partners of Gab and exert similar effects in human gastric cells [80]. Intriguingly, this concept of molecular mimicry is strongly supported by transgenic studies in *Drosophila*, demonstrating that a *cagA* transgene can rescue larval viability and photoreceptor development in mutant animals that lack DOS [84]. In addition, an epistasis analysis demonstrated that the complementation of DOS by CagA overexpression requires the expression of the SHP2 ortholog CSW [84]. Thus, these results revealed how CagA can mimic Gab/DOS proteins *in vivo*.

## 9. Conclusion 

Since the discovery of Gab docking proteins, a little more than a decade ago, it has become evident that these proteins play critical roles in a variety of physiological processes as well as in disorders including cancer, inflammation, and cardiovascular diseases. Quite recently, a genome-wide association study conducted by Tamari's group identified Gab1 as a candidate gene for adult asthma in the Japanese population [85]. Whereas the molecular mechanism underlying this association remains unclear, further studies focusing on Gab proteins will aid in elucidating the pathophysiology of this kind of bronchial asthma in the near future. Thus, the versatile functions of Gab docking proteins might extend beyond the original definition of a docking protein. Furthermore, through careful analyses of Gab docking proteins, as shown in this paper, we may be able to obtain a more detailed understanding of Gab-mediated cardiovascular diseases, cancers, and inflammation.
